# Response to Letter to the Editor Entitled “Clarifying Treatment Compliance and Future Directions in Perioperative Chemotherapy for Resectable Colorectal Liver Metastases”

**DOI:** 10.1002/ags3.70086

**Published:** 2025-09-02

**Authors:** Katsunori Shinozaki, Tsuyoshi Kobayashi, Yuji Takakura, Satoshi Ikeda, Hiroyuki Egi, Yuzo Hirata, Manabu Shimomura, Takafumi Oshiro, Takao Hinoi, Daisuke Sumitani, Masahiro Nakahara, Masanori Yoshimitsu, Naruhiko Honmyo, Junko Tanaka, Hideki Ohdan, the Hiroshima Surgical study group of Clinical Oncology (HiSCO)

**Affiliations:** ^1^ Division of Clinical Oncology Hiroshima Prefectural Hospital Hiroshima Japan; ^2^ Department of Gastroenterological and Transplant Surgery, Graduate School of Biomedical and Health Sciences Hiroshima University Hiroshima Japan; ^3^ Department of Gastroenterological and Transplant Surgery Hiroshima Prefectural Hospital Hiroshima Japan; ^4^ Department of Surgery Chugoku Rosai Hospital Kure Japan; ^5^ Department of Surgery National Hospital Organization Higashihiroshima Medical Centre Higashi‐Hiroshima Japan; ^6^ Department of Surgery JR Hiroshima Hospital Hiroshima Japan; ^7^ Department of Surgery National Hospital Organization Kure Medical Centre Kure Japan; ^8^ Department of Surgery Kure City Medical Association Hospital Kure Japan; ^9^ Department of Surgery Onomichi General Hospital Onomichi Japan; ^10^ Department of Surgery Hiroshima City Asa Hospital Hiroshima Japan; ^11^ Department of Epidemiology, Infectious Disease Control and Prevention, Institute of Biomedical and Health Sciences Hiroshima University Hiroshima Japan

**Keywords:** clinical‐malignant, colorectal, colorectal liver metastasis

1

We would like to respond to Letter to the Editor by Okuno et al. entitled “Clarifying treatment compliance and future directions in perioperative chemotherapy for resectable colorectal liver metastases” in relation to our research group's original paper, “Preoperative Versus Postoperative Chemotherapy With CAPOX Plus Bevacizumab for Resectable Colorectal Liver Metastases: A Randomized Phase II Trial (HiSCO‐01)” published in the *Annals of Gastroenterological Surgery*.

We appreciate your interest in our paper and your constructive comments.

First, in your comments, you mentioned that the reason for the low TCR in the postoperative group is still unclear. However, the consort flow diagram in figure 1 *of our original paper* shows that chemotherapy could not be started after hepatectomy in three cases (one due to a decline in PS, two due to postoperative complications), and chemotherapy was discontinued in eight cases (two due to postoperative complications, four due to adverse events associated with chemotherapy, and two due to disease progression) after five cycles or less [1]. This is the reason for the low TCR in the postoperative group. In other words, although there is no statistically significant difference between chemotherapy after hepatectomy and preoperative chemotherapy, we believe that it is highly likely to be difficult to implement.

As for your second comment, we understand the idea of selecting anti‐EGFR antibodies or bevacizumab based on molecular profiling. However, we feel that the number of colorectal cancer patients with resectable liver metastases is by no means large. The HEPATICA trial is a phase III multicenter randomized controlled trial conducted in the Netherlands regarding postoperative adjuvant therapy for patients with resected liver metastases from colorectal cancer [2]. The purpose of this study was to evaluate the efficacy (disease‐free survival, overall survival, etc.) and safety of adding bevacizumab (a molecularly targeted drug) to CAPOX (capecitabine + oxaliplatin) monotherapy as postoperative adjuvant therapy compared with CAPOX (capecitabine + oxaliplatin) monotherapy. Like ours, this study was terminated prematurely. In addition, the New EPOC study also evaluated the efficacy of adding cetuximab to chemotherapy as perioperative chemotherapy. However, an interim analysis showed shorter progression‐free survival (PFS) than expected in the chemotherapy + cetuximab group, and the study was discontinued in November 2012 [3]. For this reason, the NCCN panel recommends against the use of panitumumab and cetuximab as perioperative treatment for resectable metachronous metastatic disease. Clinical trials based on the concept of selecting biologics based on molecular profiling and combining them with chemotherapy are interesting, but I believe they would be difficult to implement in reality.

In conclusion, if a phase 3 trial is considered feasible, we will consider eight cycles of postoperative chemotherapy with CAPOX as the control group and eight cycles of preoperative chemotherapy with CAPOX + bevacizumab as the study treatment group.

## Author Contributions

K.S. and T.K.: conceptualization, investigation, writing – original draft. Y.T. and H.O.: supervision, writing – review and editing. S.I., H.E., Y.H., M.S., T.O., T.H., D.S., M.N., M.Y., N.H., and J.T.: review. All authors have read and agreed to the published version of the manuscript.

## Conflicts of Interest

Dr. Hideki Ohdan is a current Editor or Editorial Board Member of AGSurg.

## Linked Articles

This article is linked to Okuno et al. paper. To view this article, visit https://doi.org/10.1002/ags3.70062.
